# Supplementary material for: Genome-wide gene network uncover temporal and spatial changes of genes in auxin homeostasis during fruit development in strawberry (F. × ananassa)
Source: BMC Plant Biol. 2024 Sep 20;24:876. doi: 10.1186/s12870-024-05577-5 (PMC11414167; doi:10.1186/s12870-024-05577-5)
Supplement: Supplementary file 1 — Supplementary Material 1. [file 12870_2024_5577_MOESM1_ESM.pdf]

Supplemental Table 1 Representative statistics RNA-Seq quality for transcriptome analysis of achene and receptacle samples

RNA-seq sample	Total Number of Raw Read	Total Number of Average Clean Read	Average Mapped Reads	Average of Uniquely Alignment Mapping Rate (%)
Achene	754,515,524	39,798,491	32,030,949	95.6
Receptacle	797,526,052	43,861,037	34,626,525	95.3

Supplemental Table 2 Individual RNA-Seq quality results for transcriptome statistics of achene and receptacle samples at each stage of fruit development

Tissue	Name	sample	Raw reads	Total Clean Reads	Mapped Reads	Uniquely Genome Mapping (%)
Achene	S1_2	SFS_01	45,263,660	44,325,864	35,043,352	95.3
	S1_3	SFS_02	38,055,068	37,344,598	29,892,172	95.3
	S1_5	SFS_03	37,414,574	36,660,584	29,243,746	95.3
	S2_1	SFS_04	39,380,610	38,612,644	30,739,728	95.3
	S2_2	SFS_05	47,369,634	34,941,536	28,146,630	95.5
	S2_5	SFS_06	43,135,742	28,207,104	22,470,116	95.2
	S3_1	SFS_07	47,310,592	46,373,384	37,795,932	96.0
	S3_2	SFS_08	40,993,712	40,159,786	32,949,422	95.9
	S3_3	SFS_09	36,379,534	35,605,390	29,056,538	95.9
	S4_1	SFS_10	44,501,374	43,677,314	35,308,708	96.0
	S4_2	SFS_11	37,622,676	36,796,116	29,462,096	95.8
	S4_3	SFS_12	39,775,782	38,931,858	30,935,758	95.8
	S5_1	SFS_13	42,396,862	41,971,518	33,828,592	95.7
	S5_2	SFS_14	42,990,328	42,565,378	34,415,744	95.4
	S5_3	SFS_15	40,295,032	39,910,476	32,452,494	95.6
	S6_1	SFS_16	47,666,482	47,144,870	37,909,112	95.4
	S6_2	SFS_17	39,607,236	39,222,896	31,250,562	95.3
	S6_3	SFS_18	44,356,626	43,921,516	35,656,382	95.5
Receptacle	F1_1	SFS_19	46,896,542	46,439,712	35,952,508	95.4
	F1_2	SFS_20	45,721,414	45,287,676	35,110,918	95.6
	F1_3	SFS_21	45,000,136	44,473,046	35,242,252	95.3
	F2_1	SFS_22	43,708,904	43,217,140	34,109,334	95.2
	F2_2	SFS_23	43,623,310	43,148,586	34,225,420	95.3
	F2_3	SFS_24	46,617,630	46,171,926	36,342,168	95.2
	F3_1	SFS_25	45,708,024	45,201,908	35,994,676	95.1
	F3_2	SFS_26	43,786,458	43,335,508	34,398,914	95.1
	F3_3	SFS_27	39,775,888	39,374,954	31,182,498	95.1
	F4_1	SFS_28	42,588,780	42,103,296	33,450,814	95.3

F4_2	SFS_29	48,292,048	47,814,774	37,592,466	95.3
F4_3	SFS_30	41,476,064	41,066,120	32,558,040	95.4
F5_1	SFS_31	45,751,922	45,327,046	35,647,356	95.3
F5_2	SFS_32	42,019,046	41,675,318	32,014,676	95.4
F5_3	SFS_33	41,890,758	41,506,538	33,273,706	95.3
F6_1	SFS_34	45,025,180	44,551,424	35,393,794	95.1
F6_2	SFS_35	43,580,284	43,164,880	34,250,664	95.5
F6_3	SFS_36	46,063,664	45,638,808	36,537,244	95.3
