# Supplementary material for: Genome-wide gene network uncover temporal and spatial changes of genes in auxin homeostasis during fruit development in strawberry (F. × ananassa)
Source: BMC Plant Biol. 2024 Sep 20;24:876. doi: 10.1186/s12870-024-05577-5 (PMC11414167; doi:10.1186/s12870-024-05577-5)
Supplement: Supplementary file 8 — Supplementary Material 8. [file 12870_2024_5577_MOESM8_ESM.pdf]

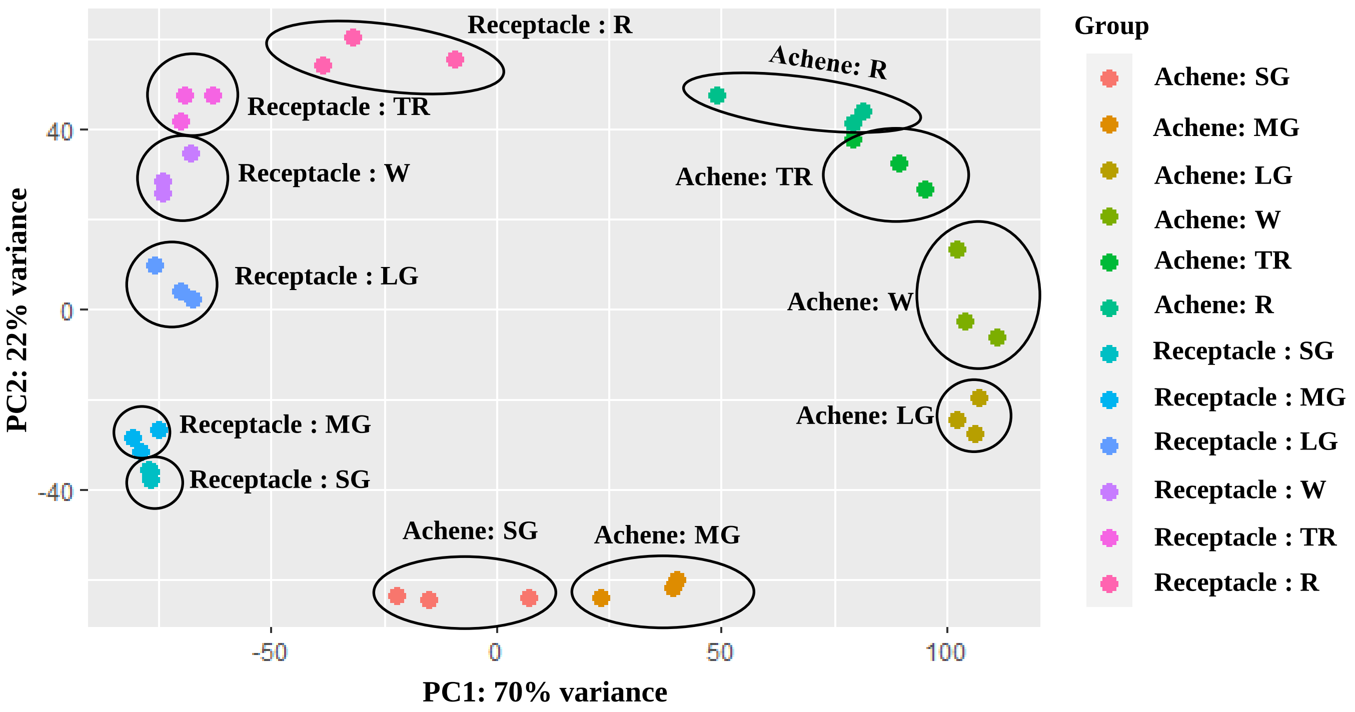

Supplemental Fig. 1. Principal component analysis (PCA) of transcriptome data from 36 samples. Each colored prototype demonstrates the distinct separation of developmental samples into different stages throughout the entire time course. Furthermore, the analysis includes 36 samples, encompassing different tissue types, namely achene and receptacle, as well as 3 biological replicates for each stage.

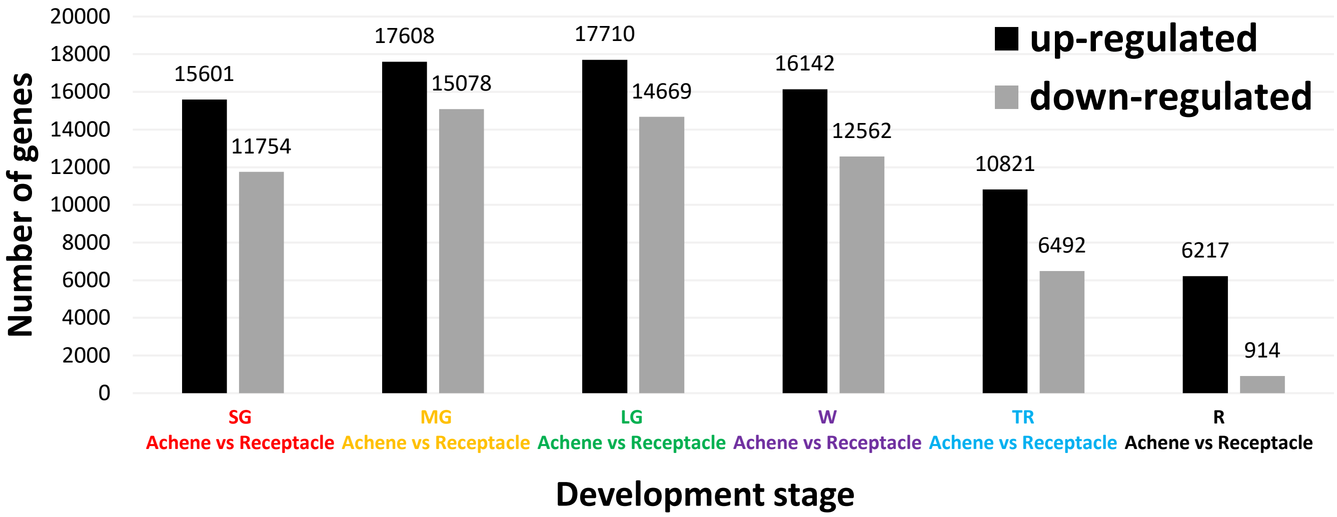

Supplemental Fig. 2. Comparison of vesicular and receptor differentially expressed genes via DESeq2

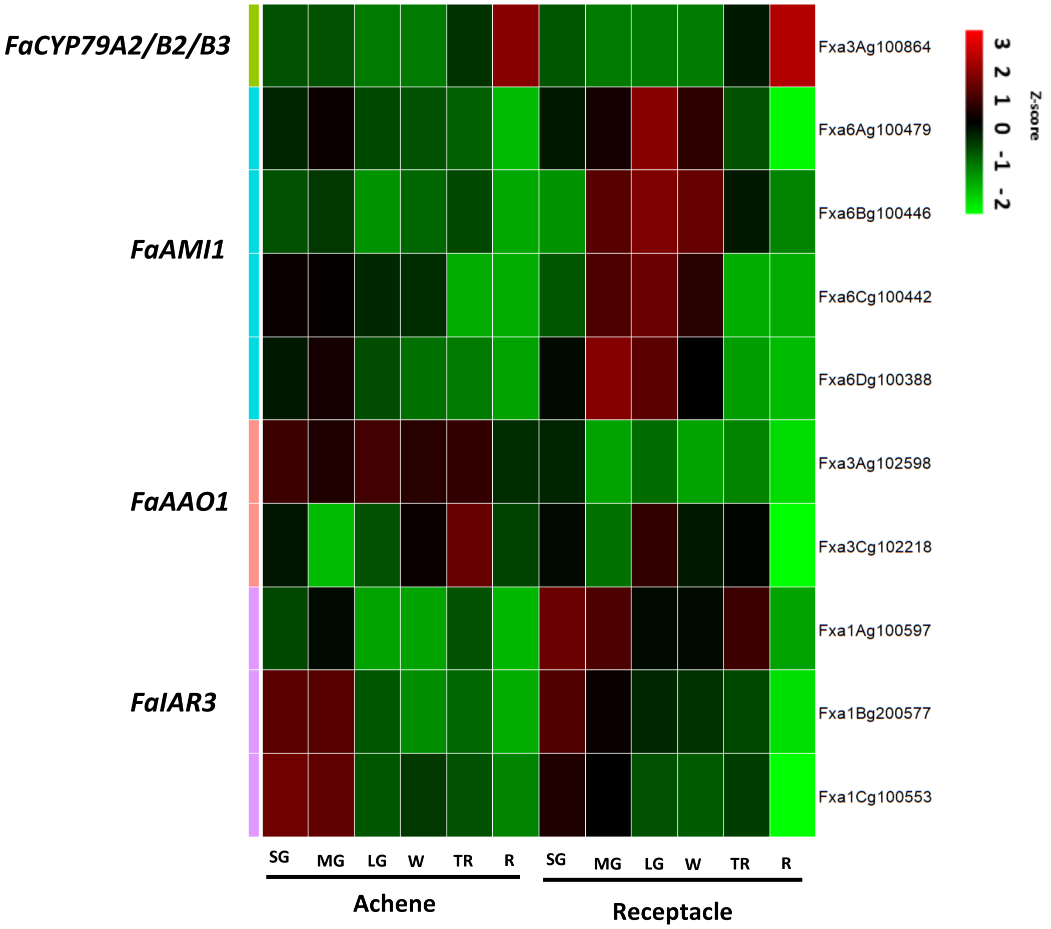

Supplemental Fig. 3 Heatmap illustrating the expression levels of genes involved in alternative auxin biosynthesis across different tissues and developmental stages in octoploid strawberries. High expression levels are indicated in red, while low expression levels are indicated in green.

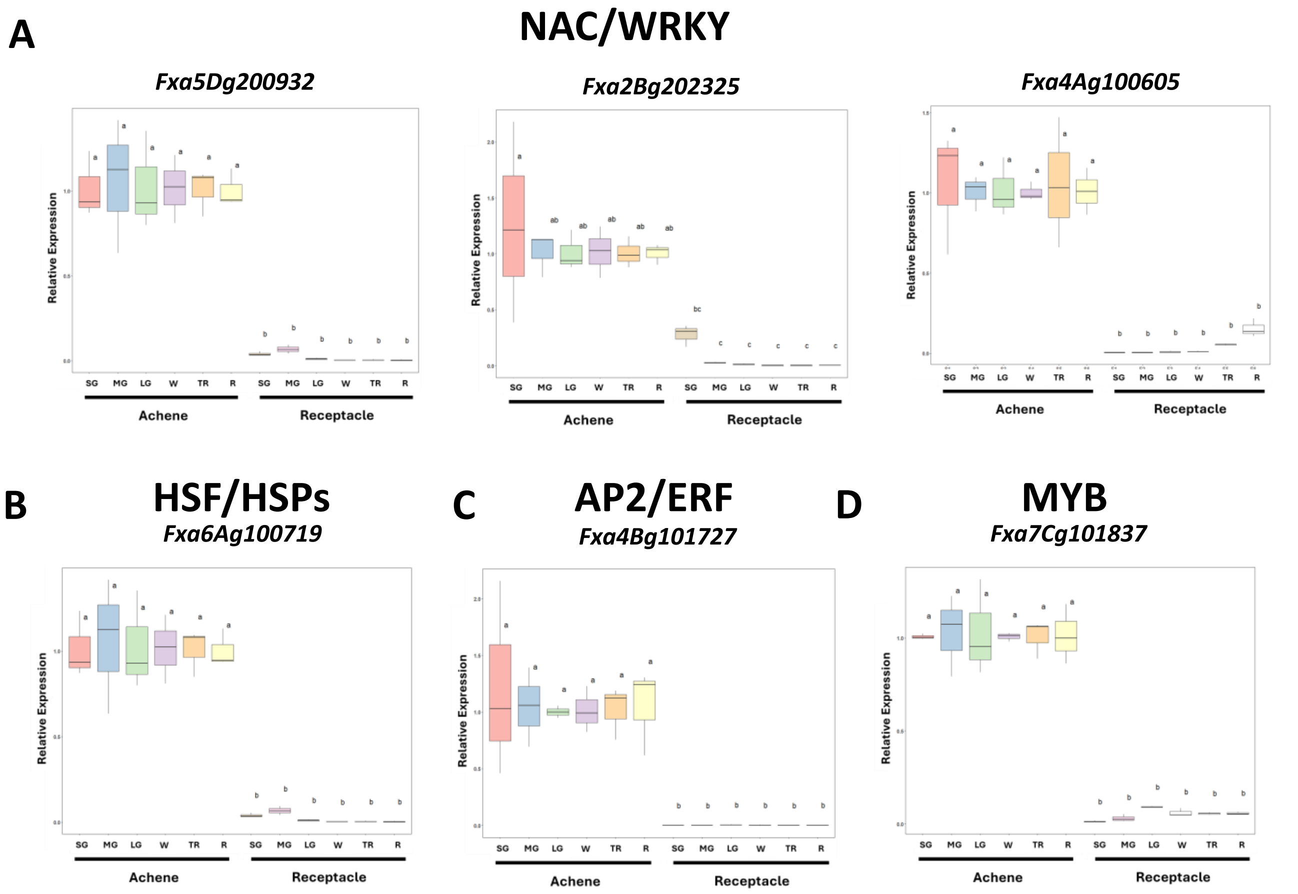

Supplemental Fig. 4 Gene expression profiling of auxin-related genes associated with transcription factors in achene and receptacle across six developmental stages

Identified auxin-related genes, each clustered with transcription factors, were analyzed for relative gene expression levels using qRT-PCR, normalized to the *FaGAPDH* gene. Expression levels were compared across six developmental stages in both achene and receptacle tissues. Statistical analysis was performed using one-way ANOVA, followed by Tukey's post-hoc test for multiple comparisons.

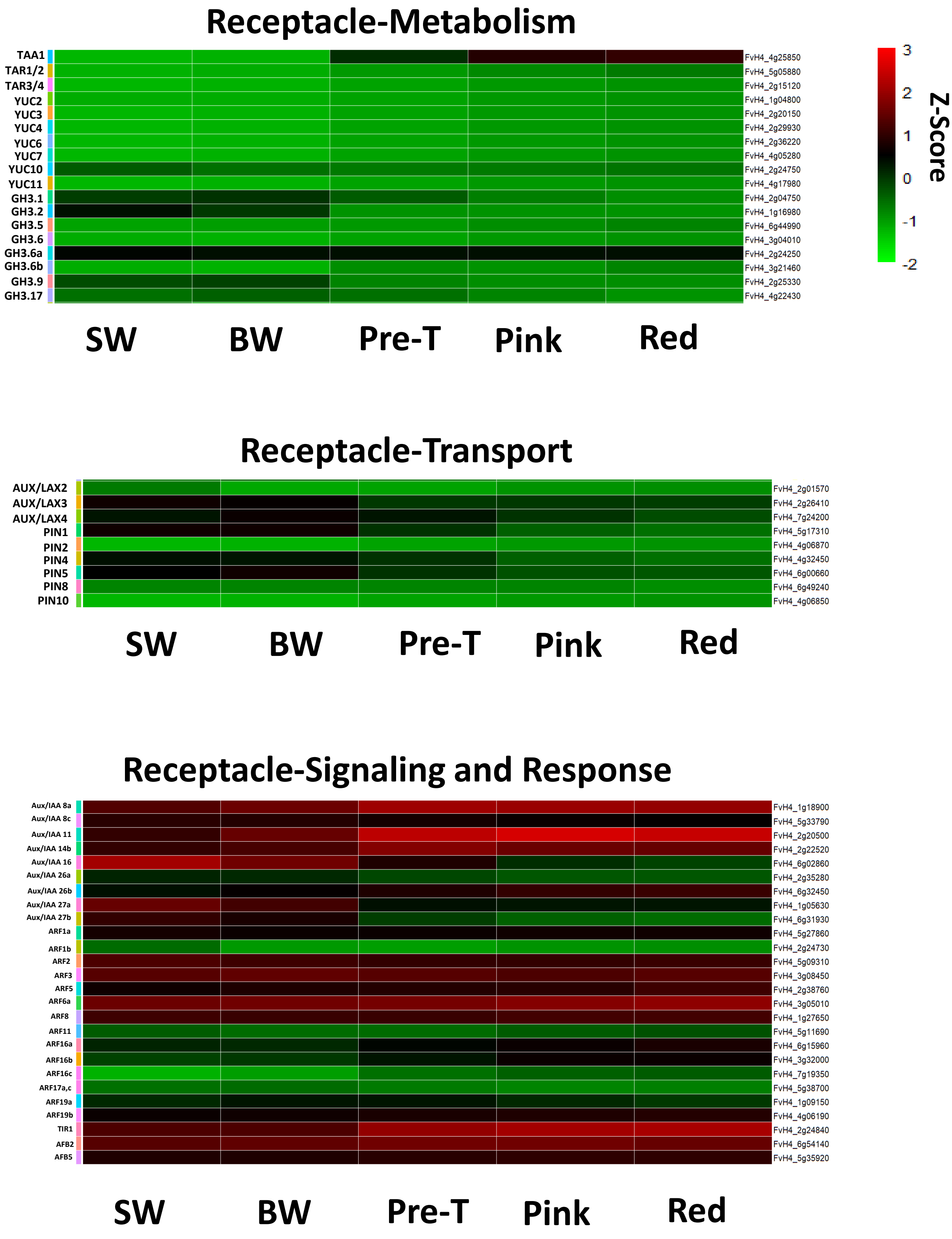

Supplemental Fig. 5 Expression Patterns of Auxin-Related Genes in *Fragaria vesca*.
Based on the Gu et al. (2019) gene expression levels were normalized to z-scores using a log2(1+FPKM) transformation and visualized using a heatmap plot with a color scale. The gradient from green to red represents gene expression values, where green indicates lower expression and red indicates higher expression. The developmental stages are Small White (SW), Big White (BW), Pre-T, Pink, and Red.
